# Paraneoplastic limbic encephalitis with associated hypothalamitis mimicking a hyperdense hypothalamic tumor: a case report

**DOI:** 10.1186/s12880-016-0113-4

**Published:** 2016-01-18

**Authors:** Vipula R. Bataduwaarachchi, Nirmali Tissera

**Affiliations:** Department of Pharmacology and Pharmacy, Faculty of Medicine University of Colombo, PO Box 271, Kynsey Road, Colombo 8, Sri Lanka; Department of Medicine, National Hospital, Ward Place, Colombo Sri Lanka

**Keywords:** Hypothalamitis, Paraneoplastic limbic encephalitis, Cranial diabetes insipidus, Noncontrast computed tomography

## Abstract

**Background:**

Paraneoplastic limbic encephalitis is an uncommon association of common malignancies such as small cell lung carcinoma, testicular teratoma, and breast carcinoma. The nonspecific nature of the clinical presentation, lack of freely available diagnostic markers, and requirement for advanced imaging techniques pose a great challenge in the diagnosis of this disease in resource-poor settings.

**Care presentation:**

A 64-year-old previously healthy Sri Lankan man was admitted to the general medical unit with subacute memory impairment regarding recent events that had occurred during the previous 3 weeks. Initial noncontrast computed tomography of the brain revealed a hyperdensity in the hypothalamic region surrounded by hypodensities extending toward the bilateral temporal lobes; these findings were consistent with a possible hypothalamic tumor with perilesional edema. The patient later developed cranial diabetes insipidus, which was further suggestive of hypothalamic disease. Interestingly, gadolinium-enhanced magnetic resonance imaging of the brain showed no such lesions; instead, it showed prominent T2-weighted signals in the inner mesial region, characteristic of encephalitis. The possibility of tuberculosis and viral encephalitis was excluded based on cerebrospinal fluid analysis results. Limbic encephalitis with predominant hypothalamitis was suspected based on the radiological pattern. Subsequent screening for underlying malignancy revealed a mass lesion in the right hilum on chest radiographs. Histological examination of the lesion showed small cell lung cancer of the “oat cell” variety.

**Conclusion:**

We suggest that the initial appearance of a hyperdensity in the hypothalamus region on noncontrast computed tomography is probably due to hyperemia caused by hypothalamitis. If hypothalamitis is predominant in a patient with paraneoplastic limbic encephalitis, magnetic resonance imaging will help to differentiate it from a hypothalamic secondary deposit. Limbic encephalitis should be considered in a patient with computed tomographic evidence of a central hyperdensity surrounded by bitemporal hypodensities. This pattern of identification will be useful for early diagnosis in resource-poor settings.

## Background

Paraneoplastic limbic encephalitis (PLE) refers to an inflammatory process localized to structures of the limbic system. PLE manifests as a subacute form of encephalitis in later adult life [1]. The occurrence of this disease has been less frequently reported in Asian than in Western countries, probably because of underdiagnosis and under-reporting. PLE is an uncommon association of common malignancies such as small cell lung carcinoma (SCLC) (50 %), testicular teratoma (20 %), and breast carcinoma (8 %); therefore it is considered as a paraneoplastic syndrome [2]. Different types of antineuronal antibodies have been isolated in the cerebrospinal fluid (CSF) of affected patients, including anti-Hu antibodies in patients with SCLC, and anti-Ta antibodies in patients with testicular teratoma [2, 3]. An autoimmune pathogenesis is suggested to play a role in the development of this rare disorder based on the presence of autoantibodies to various neuronal components in the CSF. Although the presence of these autoantibodies may help in the diagnosis, the lack of freely available diagnostic markers and the high number of patients with yet unidentified antibodies and other various tumor types pose a great diagnostic challenge.

Radiological evidence of limbic system involvement is considered to be an excellent supporting tool in the diagnosis of PLE; it helps to exclude other intracranial pathologies as well [2, 4]. A variety of imaging techniques have been used over the years, and the lack of advanced techniques such as magnetic resonance imaging (MRI) and positron emission tomography (PET) in resource-poor settings make the diagnosis more difficult. The PLE-associated mortality rate is considerably high, and treatment success depends on early detection and prompt treatment of the underlying malignancy. The main role of brain imaging in patients with PLE is differentiation between brain metastases and an associated paraneoplastic syndrome. Both of these conditions require entirely different management regimens; therefore, correct differentiation is vital. Although histological examination of brain tissue is ideal in differentiating these two entities, the highly invasive nature of the procedure has made it less popular among neurologists. Therefore, a firm diagnosis supported by imaging is of utmost importance to ensure the best possible care of the patient. Identification of a characteristic radiological pattern on imaging examination is important for proper early evaluation of these patients. We herein report a rare case of PLE associated with SCLC initially misdiagnosed as a hypothalamic tumor by noncontrast computed tomography (CT) imaging of the brain. This is the first such case reported in Sri Lanka.

## Case presentation

A previously healthy 64-year-old Sri Lankan man was admitted to the general medical unit because of progressive impairment of his memory regarding recent events that had occurred during the previous 3 weeks. The memory impairment was associated with irritability and confusion. He had been a smoker for 15 pack-years. Physical examination revealed no fever, and his Glasgow coma scale score was 12/15 with alternating irritability and drowsiness. His pupils were equal in size and reactive to light, and no significant neck stiffness was noted. Focal neurological signs were absent. The patient was not cooperative enough to conduct a proper mini-mental examination. His presentation was suggestive of delirium. Basic hematological investigations and renal and liver profiles were not suggestive of an extracranial cause.

**Fig. 1:**
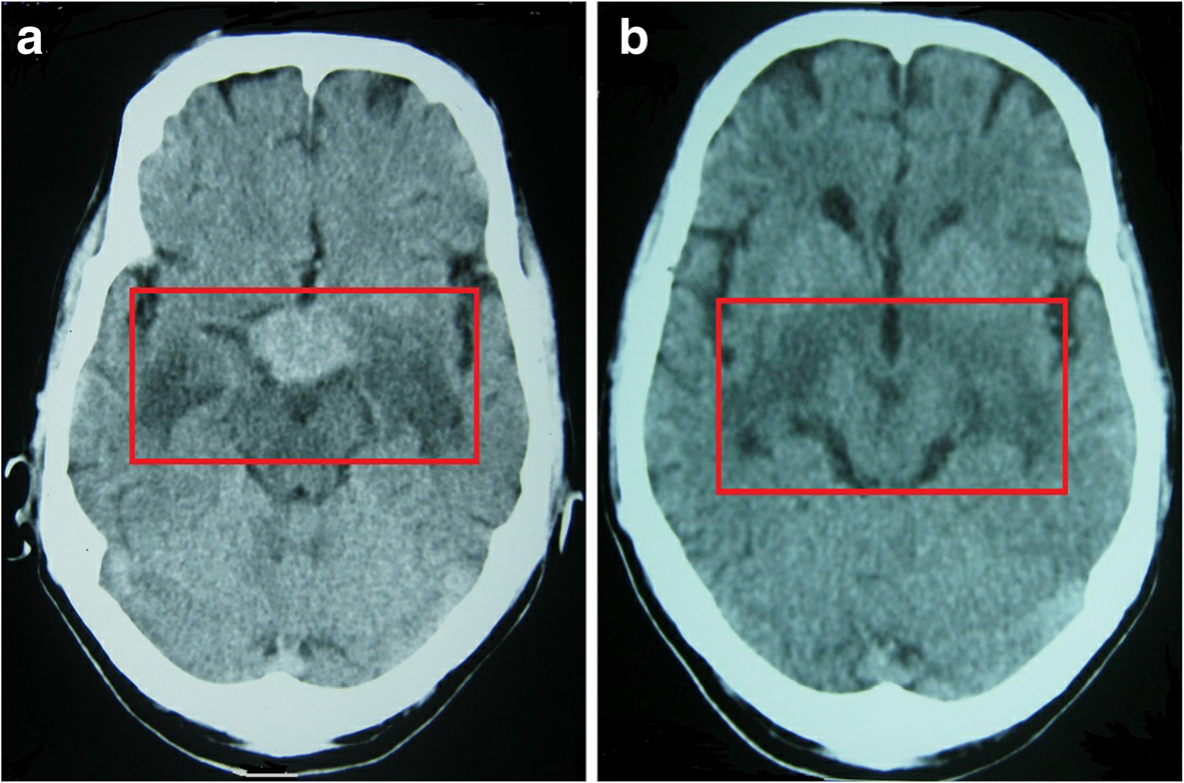
**a** Non contrast CT images. Transverse section of the brain at the thalamic level. This image showed a high density mass-like lesion in the hypothalamus region with the surrounding hypodense area in the brain. This lesion was initially reported as a mass lesion/ hemorrhage with surrounding edema. **b** Non contrast CT images. Transverse section of the brain at upper brain stem level

Before performing lumbar puncture, we carried out noncontrast CT of the brain to exclude any pathology causing increased intracranial pressure. CT revealed a hyperdensity in the hypothalamic region surrounded by hypodensities extending toward the temporal lobes bilaterally; these findings were consistent with a possible hypothalamic tumor with perilesional edema (Fig. 1a and b).

**Fig. 2:**
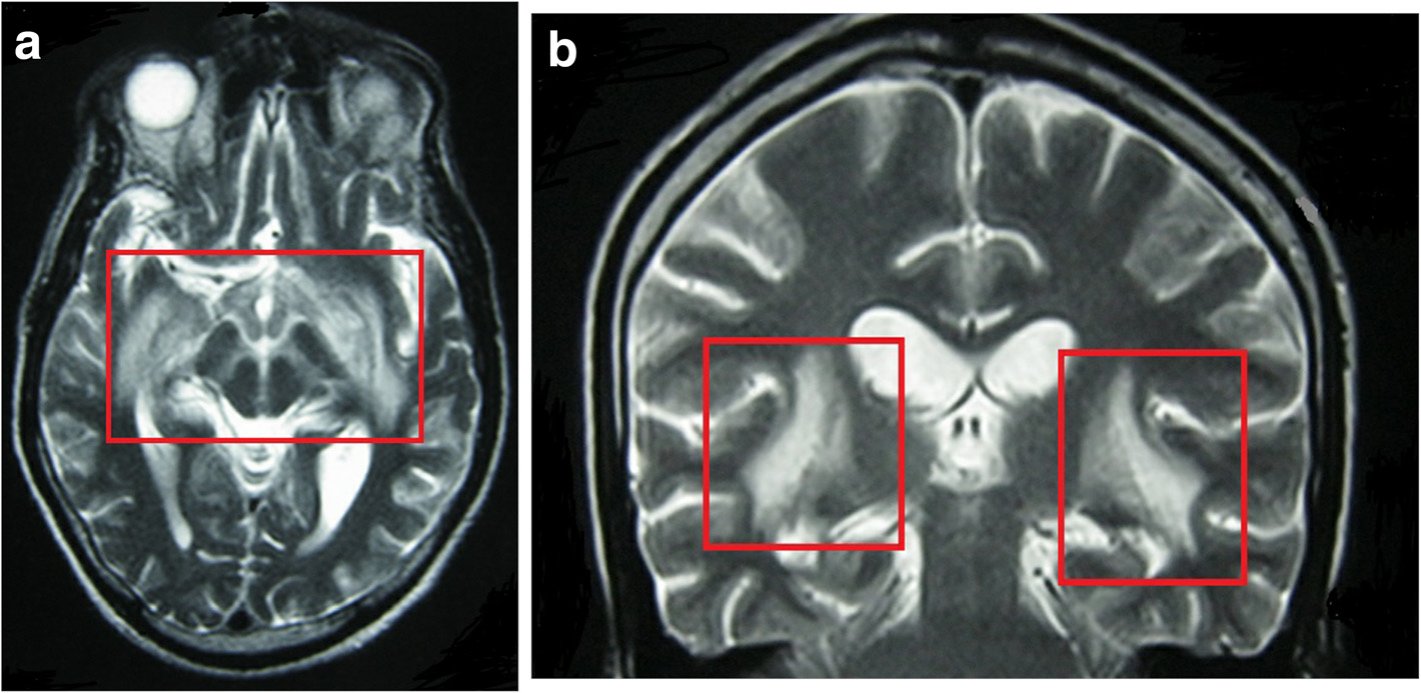
**a** Magnetic resonance (MR) images on T2 weighted images. Transverse section of the brain. Bilateral hippocampal area and mammillary bodies showed symmetrical high intensity. **b** Magnetic resonance (MR) images on T2 weighted images. Coronal section of the brain. Island and external capsule area showed symmetrical high intensity

Electroencephalography showed generalized slowing that was maximal in the bitemporal regions. On the second day of admission, his serum biochemistry parameters became abnormal with a rising serum sodium level (>160 mEq/L) and normal serum potassium level. We noticed a significant increase in his urine output (average of 5.5 L/day) and lowering of his urine osmolality (190 mOsm/kg). Based on the hypovolemic hypernatremia and low urine osmolality associated with the radiological abnormalities in the hypothalamus, we suspected underlying cranial diabetes insipidus in this patient. He was treated with rehydration and intranasal desmopressin, to which he showed only a partial response. The patient was then transferred to the intensive care unit for close monitoring. Once the patient was stable, gadolinium-enhanced MRI of the brain and brain stem was carried out. The transverse section of the brain showed increased T2-weighted signals in the deep temporal lobes, suggesting the involvement of the limbic system (Fig. 2a). The coronal section of the brain showed increased T2-weighted signals in inner mesial regions (Fig. 2b). Interestingly, the mass lesion had disappeared during the previous 3 days. Therefore, viral or autoimmune encephalitis was considered as a more probable diagnosis than a mass lesion. Lumbar puncture was performed at this point, and samples were sent for screening of viral markers (herpes simplex virus, cytomegalovirus, and Japanese encephalitis virus) and tuberculosis polymerase chain reaction; all results were negative. Cytology of the CSF was negative for any malignant cells; however, lymphocytosis was present, suggesting an underlying inflammatory process. Based on the clinical and radiological picture, PLE with associated predominant hypothalamitis was suspected in this patient. Unfortunately, autoimmune markers were not available, and the patient had financial limitations that prevented further investigation. His condition deteriorated with the development of acute kidney injury caused by severe intravascular hypovolemia due to resistant diuresis. Considering the possibility of an associated malignancy, a chest radiograph was obtained and showed a mass lesion in the right hilum. The patient was not fit enough to undergo contrast-enhanced axial CT of the thorax because of his rising serum creatinine level. However, bronchoscopy was performed, and histological examination of the hilar mass showed a SCLC of the “oat cell” variety. Unfortunately, the patient died before the oncology referral while being treated in the intensive care unit.

## Conclusions

PLE is a rare neurological syndrome characterized by personality changes, irritability, depression, seizures, memory loss, and sometimes dementia. Common solid tumors such as SCLC, testicular teratoma, and breast cancer are well known to be associated with PLE. Although the exact pathophysiological link is not obvious, the presence of antineuronal antibodies has been documented in some cases. However, the lack of specificity and availability of these markers make testing for these antibodies less helpful for diagnosis. Where these markers are unavailable, brain imaging can be used to support the diagnosis in cases of progressive neurological deterioration. Our patient was initially suspected to have a tumor of the hypothalamus with associated cranial diabetes insipidus. We suggest that the initial appearance of a hyperdensity in the hypothalamus could be due to hyperemia secondary to hypothalamitis. This is supported by previous evidence on CT perfusion images that showed a focal increase in the cerebral blood flow and shortening of the mean transit time in the bilateral hippocampi and amygdalae in a patient with encephalitis [5]. Bitemporal hypodensities on a noncontrast CT scan should always raise suspicion for encephalitis, and it is important to arrange an early MRI scan for further evaluation of such lesions. MRI can show unequivocal involvement of temporolimbic structures and helps to exclude other diagnoses [6]. Although MRI is more sensitive than CT for the detection of inflammatory brain lesions because of the higher contrast resolution, CT is still valuable when used with other supporting clinical evidence in settings where other sophisticated scan techniques are not freely available [7]. There are few reported cases of normal CT scans in patients with PLE, and precise epidemiological data are not available [8, 9]. CT evidence of a central hyperdensity surrounded by a bitemporal hyperdensity is a valuable tool with which to identify possible PLE in resource-poor settings. The role of 2-deoxy-2-[F-18] fluoro-D-glucose-positron emission tomography imaging in the clinical management of paraneoplastic neurological syndrome is evolving and thus far has shown much better results than MRI [10]. These investigations are less popular in resource-poor settings because of the lack of availability and higher cost. Hypothalamitis is a less established pathological entity. However, histological diagnosis is not practical because of the highly invasive nature of biopsy and the deep location of the hypothalamus. Several reported cases of diagnostic stereotactic biopsy have revealed inflammatory infiltrates mainly constituting lymphocytic inflammation [11–13]. Based on these findings of a common histological pattern, we suggest that highly invasive biopsy has lower priority than imaging. Other than acute paraneoplastic hypothalamitis, another variety of hypothalamitis is relapsing autoimmune hypothalamitis [14]. However, based on the present case and those described in the literature, we suggest that screening for a hidden malignancy should be an essential step in the initial evaluation of the patient. Another pathological entity that presents with cranial diabetes insipidus is infundibuloneurohypophysitis [15, 16]. Radiological differentiation of pure hypothalamitis from infundibuloneurohypophysitis is rather difficult. Importantly, this demarcation is not essential for appropriate patient management.

In summary, this case report provides useful insights regarding the radiological pattern of limbic encephalitis in noncontrast CT, which may guide physicians in the early diagnosis and prompt management of PLE.

### Consent

Written informed consent was obtained from the son on behalf of this patient for publication of this case report and any accompanying images. A copy of the written consent is available for review by the Editor-in-Chief of this journal.

## Abbreviations

CT: Computed tomography
CSF: Cerebrospinal fluid
MRI: Magnatic resonance imaging
GCS: Glasgow coma scale
HSV: Herpes simplex virus
CMV: Cytomegalovirus
JE: Japanese encephalitis
PCR: Polymerase chain reaction
EEG: Electroencephalogram

## References

[1] Brierley JB, Corsellis JAN, Hierons R, Nevin S. Subacute encephalitis of later adult life. mainly affecting the limbic areas. Brain. 1960;83:357–68.[Article]

[2] Gultekin SH, Rosenfeld MR, Voltz R, Eichen J , Posner JB, Dalmau J. Paraneoplastic limbic encephalitis: neurological symptoms, immunological findings and tumour association in 50 patients. Brain. 2000;123;1481–94. 10.1093/brain/123.7.1481 10869059

[3] Corsellis JA, Goldberg GJ, Norton AR (1968). “Limbic encephalitis” and its association with carcinoma. Brain..

[4] Graus F, Saiz A (2005). Limbic encephalitis: a probably under-recognized syndrome. Neurologia.

[5] Nonaka M, Ariyoshi N, Shonai T, Kashiwagi M, Imai T, Chiba S (2004). CT perfusion abnormalities in a case of non-herpetic acute limbic encephalitis. Rinsho Shinkeigaku.

[6] Lawn ND, Westmoreland BF, Kiely MJ, Lennon VA, Vernino S (2003). Clinical, magnetic resonance imaging, and electroencephalographic findings in paraneoplastic limbic encephalitis. Mayo Clin Proc.

[7] Weber W, Henkes H, Felber S, Jänisch W, Schaper J, Kühne D (2000). Diagnostic imaging in viral encephalitis. Radiologe.

[8] Rimmelin A, Sellal F, Morand G, Quoix E, Clouet PL, Dietemann JL. Imaging of limbic paraneoplastic encephalitis. J Radiol. 1997;78(1):73–76. 9091626

[9] Lacomis D, Khoshbin S, Schick RM. MR imaging of paraneoplastic limbic encephalitis. J Comput Assist Tomogr. 1990;14(1):115–17. 10.1097/00004728-199001000-00021 2153718

[10] Basu S, Alavi A (2008). Role of FDG-PET in the clinical management of paraneoplastic neurological syndrome: detection of the underlying malignancy and the brain PET-MRI correlates. Mol Imaging Biol.

[11] Lecube A, Francisco G, Rodríguez D, Ortega A, Codina A, Hernández C (2003). Lymphocytic hypophysitis successfully treated with azathioprine: first case report. J Neurol Neurosurg Psychiatry.

[12] Yang GQ, Lu ZH, Gu WJ, Du J, Guo QH, Wang XL (2011). Recurrent autoimmune hypophysitis successfully treated with glucocorticoids plus azathioprine: a report of three cases. Endocr J.

[13] Zhang S, Ye H, Zhang Z, Lu B, Yang Y, He M (2015). Successful diagnosis of hypothalamitis using stereotactic biopsy and treatment: a case report. Medicine (Baltimore).

[14] Wang XL, Lu JM, Yang LJ, Lü ZH, Dou JT, Mu YM (2008). A case of relapsed autoimmune hypothalamitis successfully treated with methylprednisolone and azathioprine. Neuro Endocrinol Lett.

[15] Takahashi M, Otsuka F, Miyoshi T, Ogura T, Makino H (1999). An elderly patient with transient diabetes insipidus associated with lymphocytic infundibulo-neurohypothysitis. Endocr J.

[16] Iwai Y, Yamanaka K, Yoshioka K, Okamoto Y, Sato T (1998). Report of four cases of lymphocytic infundibulo-neurohypothysitis. No Shinkei Geka.

